# Supplementary material for: Multistability, intermittency, and hybrid transitions in social contagion models on hypergraphs
Source: Nat Commun. 2023 Mar 13;14:1375. doi: 10.1038/s41467-023-37118-3 (PMC10011415; doi:10.1038/s41467-023-37118-3)
Supplement: Supplementary file 1 — Supplementary Information [file 41467_2023_37118_MOESM1_ESM.pdf]

Supplementary Information: Multistability, intermittency, and hybrid transitions in social contagion models on hypergraphs

Guilherme Ferraz de Arruda,[1] Giovanni Petri,[1,2] Pablo Martin Rodriguez,[3] and Yamir Moreno[4,5,1]

[1] *CENTAI Institute, Turin, Italy**

[2] *IMT Lucca, Lucca, Italy*

[3] *Department of Statistics, Federal University of Pernambuco (UFPE), Recife, PE, Brazil*

[4] *Institute for Biocomputation and Physics of Complex Systems (BIFI), University of Zaragoza, 50018 Zaragoza, Spain*

[5] *Department of Theoretical Physics, University of Zaragoza, 50018 Zaragoza, Spain*

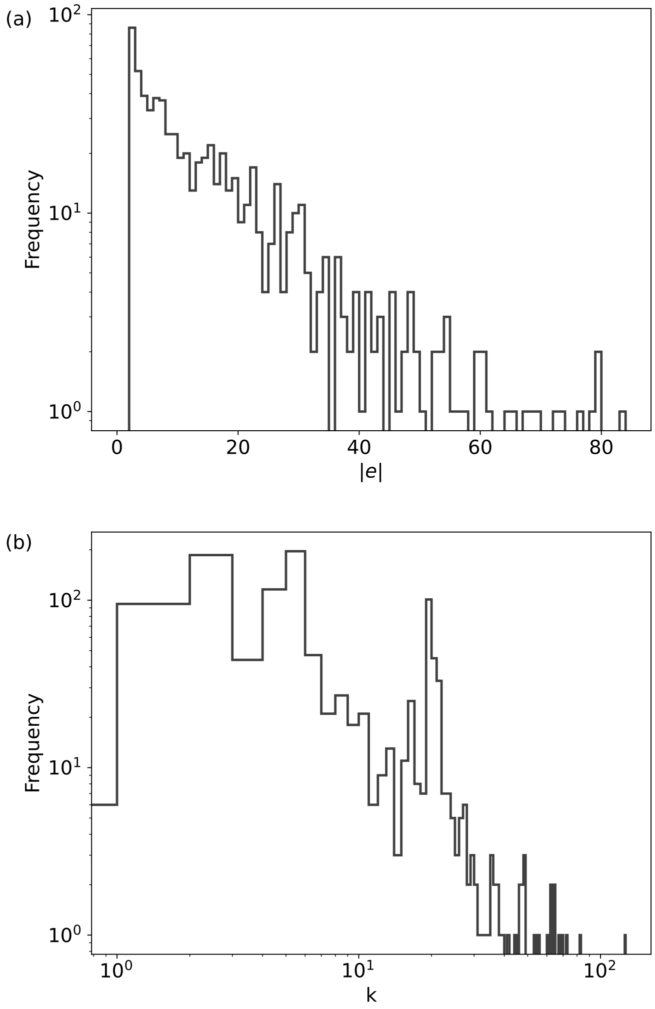

Supplementary Figure 1. Statistical characterization of the blues reviews hypergraph. In (a), we show the cardinality distribution, while in (b), we show the degree distribution.

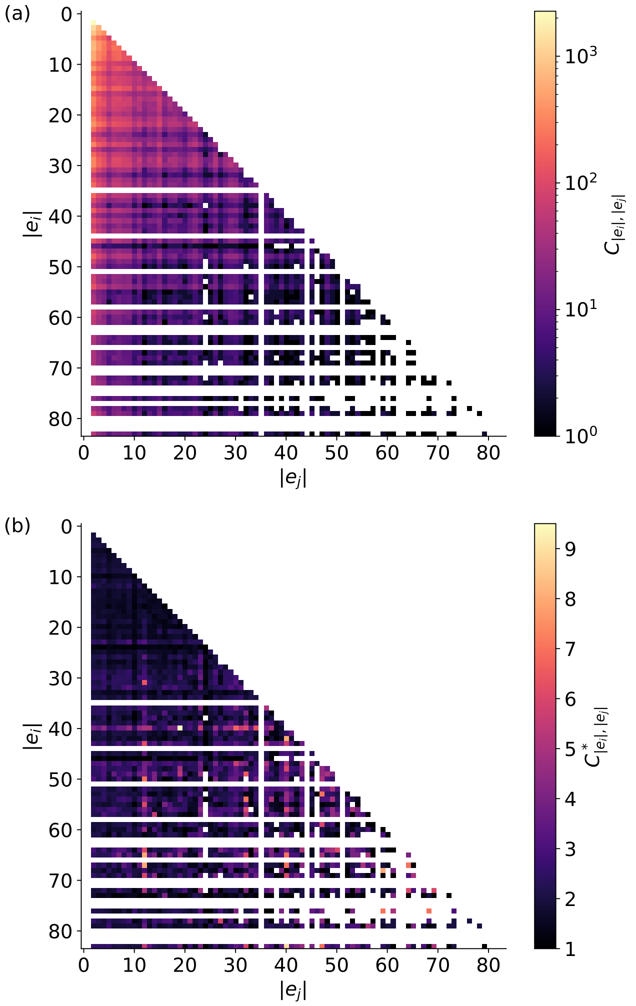

Supplementary Figure 2. Characterization of the hyperedge intersections. In (a) we show the number of hyperedges that have an intersection between hyperedges of cardinality $|e_i|$ and $|e_j|$, denoted as $C_{|e_i|,|e_j|}$. Complementary, in (b), we show the average number of nodes in the intersection between hyperedges of cardinality $|e_i|$ and $|e_j|$, $C^*_{|e_i|,|e_j|}$.

* gui.f.arruda@gmail.com

I. BLUES REVIEWS: STRUCTURAL CHARACTERIZATION

In the blues reviews hypergraph, nodes are Amazon reviewers, and hyperedges are groups of reviewers who reviewed a certain type of blues music within a month [1]. This dataset is available at [2]. This hypergraph has $N = 1106$ nodes and 694 hyperedges and its maximum cardinality is $\max(|e_j|) = 83$. In this dataset, the pairwise interactions are sparse, which alone would form a giant component of only 24 nodes. However, by accounting for all the hyperedges, the giant component of the hypergraph has $N = 1106$ nodes. In our experiments, we did not allow for repeated hyperedges.

First, we characterize the blues reviews hypergraph in terms of its cardinality and degree distributions, presented in Fig. 1. in summary, the degree distribution has an average of $\langle k \rangle \approx 9.4$ with a standard deviation of std$(k) \approx 10.7$, while the cardinality distribution has $\langle |e| \rangle \approx 15.1$ and std$(|e|) \approx 14.7$.

To characterize the intersections between hyperedges, we consider the number of hyperedges that have an intersection between hyperedges of cardinality $|e_i|$ and $|e_j|$, formally defined as

$$C_{|e_i|,|e_j|} = \sum_{e_i, e_j \in \mathcal{E}, i \neq j} \mathbb{1}_{\{e_i \cap e_j \neq \emptyset\}}. \quad (1)$$

Complementary, we also consider the average number of nodes in the intersection between hyperedges of cardinality $|e_i|$ and $|e_j|$, formally defined as

$$C^*_{|e_i|,|e_j|} = \frac{\sum_{e_i, e_j \in \mathcal{E}, i \neq j} B_{ij}}{C_{|e_i|,|e_j|}}, \quad (2)$$

where $B_{ij} = \mathcal{I}^T \mathcal{I}$ is the number of nodes in the intersection of hyperedges i and j, i.e., $|e_i \cap e_j|$, and $\mathcal{I} \in \mathbb{R}^{n \times m}$.

In Fig. 2 (a) and (b), we show $C_{|e_i|,|e_j|}$ and $C^*_{|e_i|,|e_j|}$, respectively. We notice that most intersections happen in small cardinality hyperedges, as shown in the top left of Fig. 2 (a). Moreover, we noticed that the average intersections are reasonably small. So naturally, as the sizes of both hyperedges increase, the intersection can potentially be larger. Although this pattern is indeed observed, see Fig. 2 (b), the average intersections are still reasonably small, as seen in the same figure. Note that the maximum average intersection is around 9.

We remark that the role of hyperedge intersections is also reported in [3], where a version of the SIR model in hypergraphs is used in the analysis. Ref. [3] focuses on defining and extracting components that restrict the minimum hyperedge intersection. However, here, we focus on the statistical characterization of individual hyperedge intersections without constraints regarding the connectedness of the system.

II. BLUES REVIEWS: RANDOMIZED HYPERGRAPHS

In the main text, we reported the behavior of a randomized version of the blues review hypergraph. Here, we report 30 additional simulations for different randomizations of the same structure, showing that the qualitative behavior is the same, i.e., a single discontinuous transition.

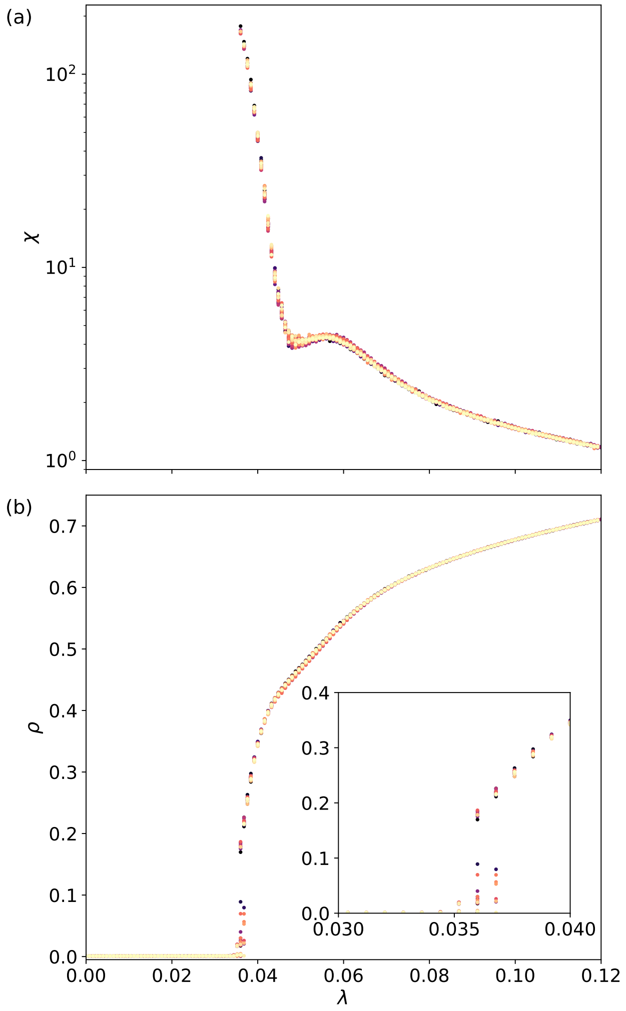

Supplementary Figure 3. Monte Carlo simulations for the social contagion model in randomized versions of the blues reviews hypergraph. In (a), we show the susceptibility curves, while in (b), we show the order parameter. In the inset of (b), we emphasize the discontinuous transition.

We relied on the same method presented in the main text to generate a randomized hypergraph. Precisely, we used the vertex-labeled hypergraph configuration model presented in [4] (Algorithm 2 in [4] and code from [5]) after 10^7 rewirings.

Fig. 3 shows our social contagion model's QS Monte Carlo simulations in 30 different randomized versions of the blues reviews hypergraph. First, we observe that all the simulations are qualitatively equivalent, presenting a single discontinuous transition. However, the position of such a transition changes from simulation to simulation. This effect is evidenced in the inset of Fig. 3 (b). Moreover, in some simulations and near the transition, we find some points between the upper and lower branch. However, more than this is needed to characterize a

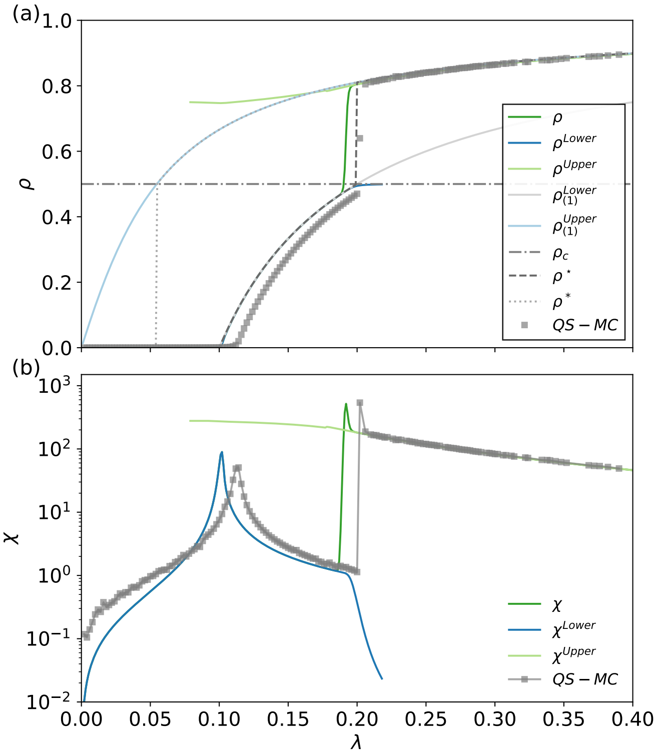

Supplementary Figure 4. Comparison between exact, first-order approximation and Monte Carlo simulations for the hyperblob. In this case, we have $N = 1000$ nodes, the average degree of the low-order structure is $\langle k \rangle = 10$. Dynamically, we fixed $\delta = 1$ and $\lambda^*(|e_j|) = \log_2(|e_j|)$.

continuous transition as the susceptibility curves still present the divergence behavior as opposed to the continuous peak expected in the continuous transition (see Fig. 3 (a)). Indeed, the cloud of points in the inset in Fig. 3 (b) is most likely related to simulations that moved from one branch to the other due to a stochastic fluctuation.

Despite the critical behavior, we also observe that the variance in the supercritical regime is visually very small. Interestingly, we also obtained a susceptibility peak in all the simulations after the transition (near the region $0.05 \leq \lambda \leq 0.07$). This peak is associated with a change in the derivative of the ρ curve.

III. ADDITIONAL HYPERBLOB ANALYSIS AND COMMENTS

To complement our analysis of hybrid phase transitions in the hyperblob structure, here, we compare the exact solution, presented in the main text, with the first-order approximation, presented in [6], and the Monte Carlo simulations using the QS algorithm. The results of these experiments are presented in Fig. 4. First, we notice that the first-order approximation is reasonable for a wide range of parameters, especially when compared to the exact equations. Next, there is a clear shift between the exact model and Monte Carlo simulations. Interestingly, this shift is also observed in the low λ regime, where we effectively have an SIS process. These results suggest that the observed displacement is related to our exact equations assuming an annealed system (the number of connections is fixed, but the neighbors are not). At the same time, the simulations are quenched (the whole structure is fixed). This observation aligns with the errors observed for the SIS in networks [7, 8].

In summary, we have evidence that the first-order approximation presented in [6] is accurate. Most importantly, from Fig. 4, we have evidence that the second peak of susceptibility is indeed expected, complementing the analytical and numerical experiments in the main text.

IV. ADDITIONAL ANALYSIS OF THE TWO COMMUNITY NUMERICAL SIMULATIONS

Complementary to the numerical simulations in the main text, here we report the results of our social contagion model on the artificial structure with two communities, however, changing the parameter μ that controls the average cardinality, which follows an exponential distribution. Differently from the main text, here, we used three initial conditions in our numerical simulations: (i) the dense community is active while the sparse is not, (ii) the sparse community is active while the dense is not, and (iii) every node is active. We highlight that in the main text, we followed random configurations varying the global average, while here, we use our knowledge of the system and its mesoscale organization to speed up the simulations. Finally, note that other branches might also exist, but this would not alter the interpretation of our results.

In Fig. 6, we show the susceptibility and the order parameter for our artificial structure with two communities as changing the parameter μ of the exponential distribution of cardinalities. We explored $\mu = 4, 8, 16, 32$, which effectively changes the average cardinality of these hypergraphs. For lower cardinalities, $\mu = 4$, in Fig. 6 (a) and (b), we observe that the discontinuous transitions are present for all the tested initial conditions. The main difference is the length of the jump. When the initial condition includes only the sparser community, the jump will be from the absorbing state to the state where the whole hypergraph is active. In other words, we do not have a separate branch relating to the case in which the sparser community alone is active. The case of $\mu = 8$, in Fig. 6 (c) and (d), is similar to the one reported in the main text. The observed differences are related to the different initial conditions employed. Despite that, the analysis is the same. As μ increases, we observe that the second transition has a continuous change from one branch to the other. However, similarly to the main text, this is also due to a bimodal distribution of states (result not shown). It is easier to see this result in the case of $\mu = 32$ in Fig. 6 (g) and (h), where the transition is smoother than for the other values of μ.

The dynamical pattern reported here is the same as the one observed in the main text for the number of bridges. However, here, the number of bridge hyperedges is fixed, while the number of nodes in each hyperedge is changing as a func-

tion of μ. Moreover, the mechanism behind this behavior is slightly different. When the bridges are small (low cardinality), if one community is active, the bridges are still unable to activate the other community. They might activate subsets of nodes but are unable to trigger the activation of the whole community. Thus, the activation process is delayed (in λ), implying that when the second community is activated, λ is high enough to keep the activity without intermittency. On the other hand, for bigger bridges (higher cardinality), the active community is able to activate the bridges, which are able to activate the other community. However, λ is not high enough to sustain the activity in this community. Thus, we observe intermittency, where stochastic fluctuations activate and deactivate the sparser community. At a mesoscale, we observe a bimodal distribution of states. At the same time, at a macroscale, we observe a peak on the susceptibility curve (for an example, please see Fig. 6 (g) and (h) near $\lambda \approx 0.015$).

In summary, the observed behavior is compatible with what was reported in the main text, where we changed the number of hyperedges, thus increasing the number of bridges and observing that the transition changed from discontinuous to continuous. Here, a similar effect is also observed, but due to an increase in the sizes of the hyperedges. Effectively, even if we have the same number of bridges, as one community alone (the denser one) is able to activate the bridge hyperedges, it might be able to propagate the dynamics to the other community (the sparser one). Thus, in summary, a similar pattern of multistability and intermittency is also observed as a function of the "size" of the bridges, complementing the results in the main text.

V. A FOUR-COMMUNITY HYPERGRAPH EXAMPLE

Aiming to strengthen our arguments about the relationship between multistability and intermittency concerning community structure, we propose an extension of the numerical simulations in the main text. Here we show that, for a well-defined community structure, each community is able to generate a level of activity and, consequently, a transition in the phase diagram. To show that, we create a hypergraph that follows a similar model as the artificial one presented in the main text, however, with four communities. The algorithm is described as follows. First, we generate communities one and two, the first containing $m_1 = 2000$ hyperedges and the second with $m_2 = 1000$ hyperedges. They are connected with $m_{1,2} = 125$ hyperedges. Next, we generate communities three and four, the third containing $m_3 = 500$ hyperedges, while the fourth with $m_4 = 250$ hyperedges. This two communities are also connected with $m_{3,4} = 125$ hyperedges. In this case, we extract a uniform number from $\ell \in [1, |e_j|)$, where ℓ is the number of nodes in one community and $\ell - |e_j|$ will be in the other community, similarly to the model described in the main text. Finally, we add $m_{2,3} = 125$ hyperedges connecting the second and third communities. The adjacency matrix of this hypergraph is shown in Fig. 5.

In Fig. 7, we show the dynamical behavior of our four communities model as a function of λ for different critical-mass

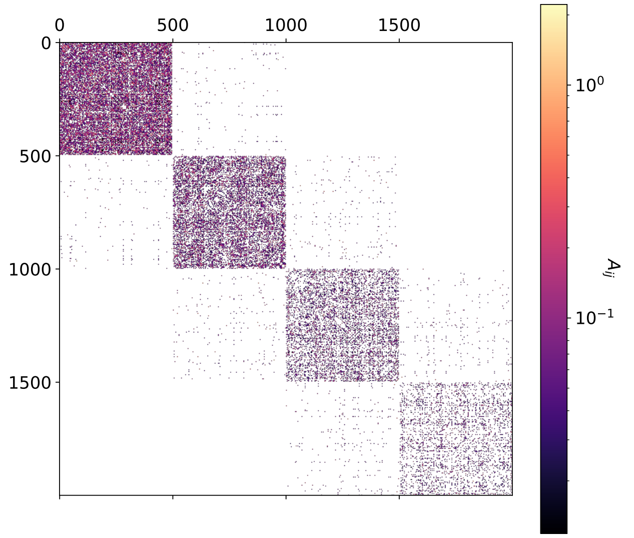

Supplementary Figure 5. Adjacency matrix with four communities using our algorithm. The number of hyperedges in each community are $n_e^1 = 2000$, $n_e^2 = 1000$, $n_e^3 = 500$, and $n_e^4 = 250$. Adjacent communities are also connected by $n_e^{1,2} = n_e^{2,3} = n_e^{3,4} = 125$. The cardinalities are exponentially distributed, i.e., $P(|e|) \sim \exp(-\mu|e|)$, with $\mu = 8$.

thresholds. We explored the parameter space using six different initial conditions: (i) X_1 all the nodes are active, (ii) X_2 only the first community is active, (ii) X_3 the second community is activated, (iv) X_4 the third community is active, (v) X_5 the fourth community is active, and (vi) X_6 the second and third communities are active. When not mentioned, the nodes are inactive.

Moreover, we vary the critical-mass parameter Θ^*, aiming to take advantage of the differences in sparsity between communities to facilitate our analysis. In this case, we notice that our notion of sparsity and the concept of bridges also depend on this parameter. Intuitively, for lower values of Θ^*, it is easier to activate the hyperedge. Thus fewer hyperedges are necessary to sustain the activity. However, the converse is also true, where for higher values of Θ^*, more hyperedges are needed to sustain the activity. Furthermore, from a dynamical viewpoint, the same interplay between multistability in well-separated communities to intermittent behavior in less well-defined communities is also observed here. Note that these results align with the results presented in the main text and Sec. IV.

Interestingly, we also note that, given our construction of a hypergraph, we can compare how the process spreads from different densities. Note that the ration between the number of hyperedges in adjacent communities is constant, i.e., $\frac{m_1}{m_2} = \frac{m_2}{m_3} = \frac{m_3}{m_4} = 2$. However, the number of hyperedges between these communities is constant, $m_{1,2} = m_{2,3} = m_{3,4} = 125$. Thus, locally, we have different sparsity levels inside the same hypergraph. Dynamically, as we reduce Θ^*, the interplay between inter and intra-community densities allow some transitions to shift from discontinuous to intermittent behavior.

For instance, by comparing Fig. 7 (a) and (b) for $\Theta^* = 0.5$ with Fig. 7 (e) and (f) for $\Theta^* = 0.3$, we see this type of shifting, where some transitions are still discontinuous while others show the intermittent pattern (continuous peak of susceptibility). Also, in the limiting case, Fig. 7 (i) and (j) for $\Theta^* = 0.1$, all the transitions follow that pattern with multiple continuous peaks of susceptibility.

To conclude, this numerical simulations shows that, under certain restrictions, we might be able to design structures with a determined dynamical behavior. For instance, we can create a hypergraph with a pre-determined number of branches by controlling the number, densities, and number of hyperedges between communities. Additionally, one might use the dynamics to find communities in a given hypergraph (a labeling problem). This experiment might not be scalable, but it is conceptually possible and can open a path for additional research.

Supplementary References

[1] J. Ni, J. Li, and J. McAuley, Justifying recommendations using distantly-labeled reviews and fine-grained aspects, in *Proceedings of the 2019 Conference on Empirical Methods in Natural Language Processing and the 9th International Joint Conference on Natural Language Processing (EMNLP-IJCNLP)* (2019) pp. 188–197.

[2] cat-edge-music-blues-reviews, `https://www.cs.cornell.edu/~arb/data/cat-edge-music-blues-reviews/`, accessed: 2021-04-25.

[3] J.-H. Kim and K. I. Goh, Higher-order components in hypergraphs (2022), arXiv:2208.05718 [physics.soc-ph].

[4] P. S. Chodrow, Configuration models of random hypergraphs, Journal of Complex Networks **8**, 10.1093/comnet/cnaa018 (2020), cnaa018.

[5] Configuration models of random hypergraphs, `https://github.com/PhilChodrow/hypergraph`, accessed: 2022-09-12.

[6] G. F. de Arruda, G. Petri, and Y. Moreno, Social contagion models on hypergraphs, Phys. Rev. Research **2**, 023032 (2020).

[7] A. S. Mata and S. C. Ferreira, Pair quenched mean-field theory for the susceptible-infected-susceptible model on complex networks, EPL (Europhysics Letters) **103**, 48003 (2013).

[8] G. F. de Arruda, F. A. Rodrigues, and Y. Moreno, Fundamentals of spreading processes in single and multilayer complex networks, Physics Reports **756**, 1 (2018).

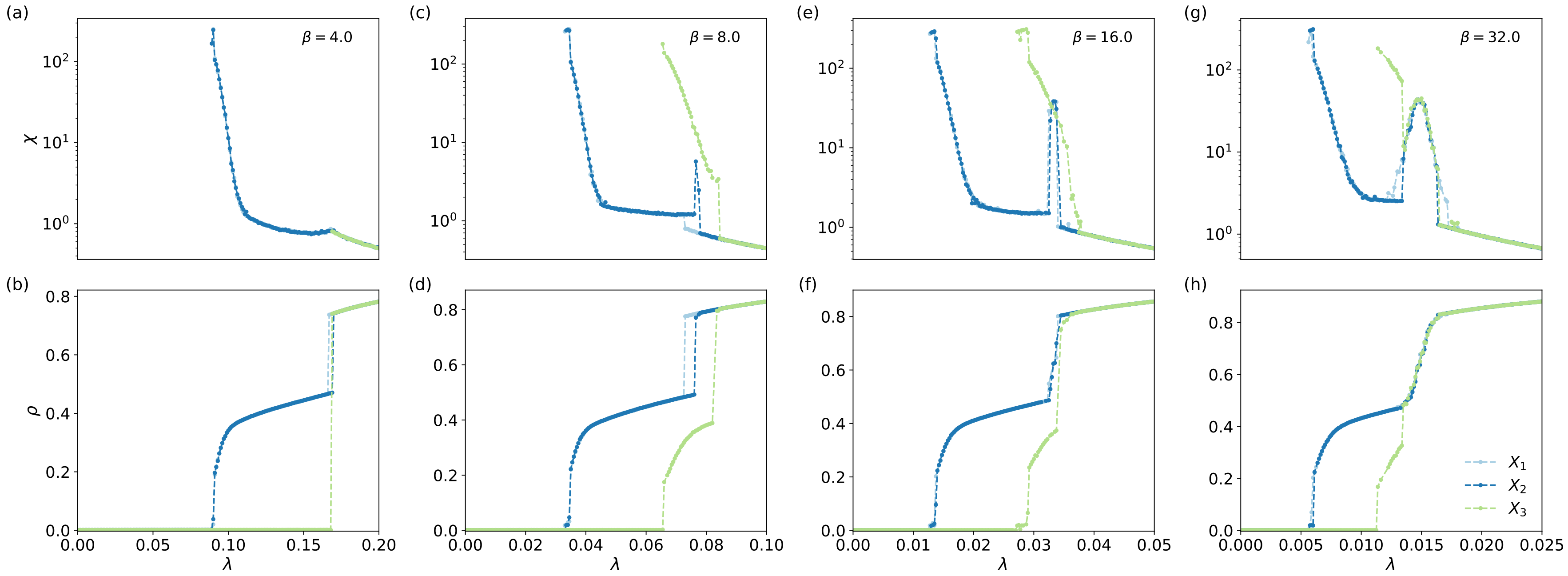

Supplementary Figure 6. Social contagion model on the two community artificial hypergraph. Here, we fix the number of nodes and hyperedges but change the average cardinality, which follows an exponential distribution $P(|e|) \sim \exp(-\mu|e|)$. Also, we fixed $\lambda^*(|e_j|) = \log_2(|e_j|)$ and $\delta = 1$. On the top panels, we show the susceptibility, while on the lower panels, we show the order parameter. In (a) and (b) have $\mu = 4$, in (c) and (d) $\mu = 8$, in (e) and (f) $\mu = 16$, while in (g) and (h) $\mu = 32$. The colors correspond to three different initial conditions that are the same in all the experiments. In X_1, all the nodes are active at $t = 0$. In X_2, the nodes in the denser community are active, and the nodes in the sparser community are inactive. In contrast, in X_3, we have the opposite, where the nodes in the sparser community are active, and the nodes in the denser community are inactive.

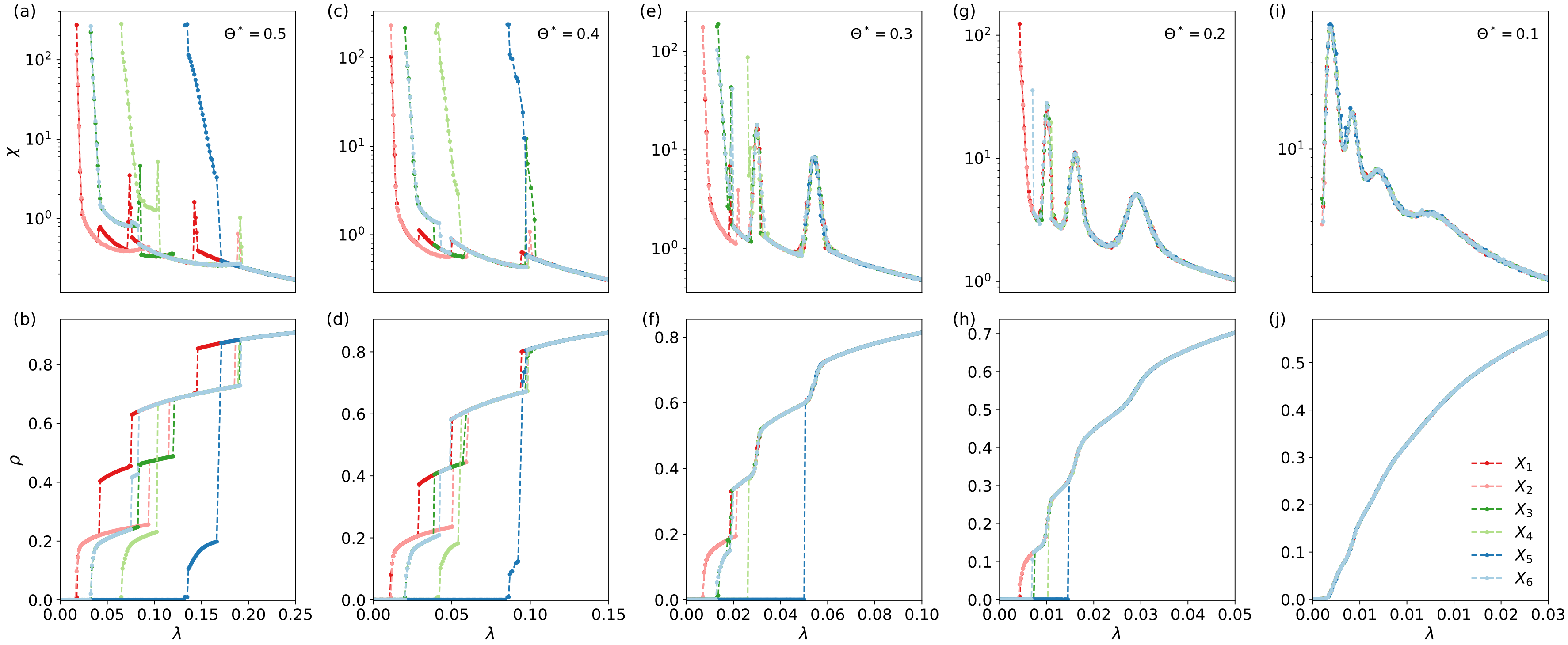

Supplementary Figure 7. Social contagion model on the four community artificial hypergraph. We fix the number of nodes, hyperedges and $\mu =$, which defines the exponential distribution $P(|e|) \sim \exp(-\mu|e|)$. Here, we evaluate the impact of the critical-mass threshold by changing Θ^* and for a fixed structure. Also, we fixed $\lambda^*(|e_j|) = \log_2(|e_j|)$ and $\delta = 1$. On the top panels, we show the susceptibility, while on the lower panels, we show the order parameter. In (a) and (b) $\Theta^* = 0.5$, in (c) and (d) $\Theta^* = 0.4$, in (e) and (f) $\Theta^* = 0.3$, in (g) and (h) $\Theta^* = 0.2$, while (i) and (j) $\Theta^* = 0.1$. The colors correspond to three different initial conditions that are the same in all the experiments. At $t = 0$, in X_1, all the nodes are active. In X_2, only the first community is active. In X_3, the second community is activated. In X_4, the third community is active. In X_5, the fourth community is active, and in X_6, the second and third communities are active. Note that, when not mentioned, the nodes are inactive.
